# Corticosterone mediates electroacupuncture-produced anti-edema in a rat model of inflammation

**DOI:** 10.1186/1472-6882-7-27

**Published:** 2007-08-14

**Authors:** Aihui Li, Rui-Xin Zhang, Yi Wang, Haiqing Zhang, Ke Ren, Brian M Berman, Ming Tan, Lixing Lao

**Affiliations:** 1 Center for Integrative Medicine, School of Medicine, University of Maryland, Baltimore, MD 21201 USA; 2 Shanghai University of Traditional Chinese Medicine, Yueyang Affiliated Hospital, Shanghai, China; 3 Dept. of Biomedical Sciences, Dental School, University of Maryland, Baltimore, MD 21201 USA; 4 Division of Biostatistics, University of Maryland Greenebaum Cancer Center, Baltimore, MD 21201 USA

## Abstract

**Background:**

Electroacupuncture (EA) has been reported to produce anti-edema and anti-hyperalgesia effects on inflammatory disease. However, the mechanisms are not clear. The present study investigated the biochemical mechanisms of EA anti-inflammation in a rat model.

**Methods:**

Three experiments were conducted on male Sprague-Dawley rats (n = 7–8/per group). Inflammation was induced by injecting complete Freund's adjuvant (CFA) subcutaneously into the plantar surface of one hind paw. Experiment 1 measured plasma corticosterone (CORT) levels to see if EA regulates CORT secretion. Experiment 2 studied the effects of the adrenal gland on the therapeutic actions of EA using adrenalectomy (ADX) rats. Experiment 3 determined whether a prototypical glucocorticoid receptor antagonist, RU486, affects EA anti-edema. EA treatment, 10 Hz at 3 mA and 0.1 ms pulse width, was given twice, for 20 min each, once immediately after CFA administration and again 2 h post-CFA. Plasma CORT levels, paw thickness, indicative of the intensity of inflammation, and paw withdrawal latency (PWL) were measured 2 h and 5 h after the CFA injection.

**Results:**

EA significantly increased plasma corticosterone levels 2 h (5 folds) and 5 h (10 folds) after CFA administration compared to sham EA control, but EA alone in naive rats and CFA alone did not induce significant increases in corticosterone. Adrenalectomy blocked EA-produced anti-edema, but not EA anti-hyperalgesia. RU486 (15 μl, 15 μg/μl), a prototypical glucocorticoid receptor antagonist, also prevented EA anti-edema.

**Conclusion:**

The data demonstrate that EA activates the adrenals to increase plasma corticosterone levels and suppress edema and suggest that EA effects differ in healthy subjects and in those with pathologies.

## 1. Background

Non-steroidal anti-inflammatory drugs (NSAIDs) and recently developed cyclooxygenase-2 (Cox-2) inhibitors are commonly used for treating inflammatory diseases such as arthritis. They are, however, associated with adverse effects such as gastrointestinal disturbances and cardiovascular risks [1-4]. Acupuncture, a therapeutic modality with few or no adverse effects, has been used in China and other Asian countries for thousands of years to treat a variety of conditions, including inflammatory disease [5,6]. Clinical trials show that electroacupuncture (EA) has beneficial effects in patients with various inflammatory diseases [7]. A recent clinical trial reported that EA significantly alleviated the symptoms of patients with knee osteoarthritis (OA) compared to sham acupuncture control during a long follow-up period of 26 weeks [8]. We recently demonstrated that EA significantly inhibits complete Freund's adjuvant (CFA)-induced hind paw inflammation and hyperalgesia in a rat model [9,10]. Despite this evidence, the underlying mechanisms of acupuncture are still not understood.

Previous studies on uninjured animals reported that acupuncture increases adrenocorticotropic hormone (ACTH) [11,12] and glucocorticoid [13-15]. Since adrenal glands secrete glucocorticoids such as cortisol in humans [13]and horses [14] and corticosterone (CORT) in rabbits [15], these studies suggest that EA may activate the adrenals to increase glucocorticoid secretion, leading to suppression of inflammatory responses.

However, uninjured models do not mimic the chronic pathological conditions seen clinically. For example, chronic stress sensitize hypothalamic-pituitary-adrenal axis (HPA) response to acute stress [16,17]. Interleukin-1 administration enhances HPA responses to foot shock in rats [18]. Further, it has been demonstrated that EA may produces differential effects under healthy and pathological conditions [19]. Thus, we used a CFA-inflamed rat model to test the hypothesis that EA increases glucocorticoid secretion to ameliorate inflammation and hyperalgesia.

## 2. Methods

### 2.1 Animal preparation

Male Sprague-Dawley rats weighing 280–350 g (Harlan, Indianapolis, IN) were kept under controlled conditions (22°C, relative humidity 40%–60%, 12-hour alternate light-dark cycles, food and water *ad libitum*). The animal protocols were approved by the Institutional Animal Care and Use Committee (IACUC) at the University of Maryland School of Medicine.

### 2.2 Experimental design

Three experiments were conducted. In Experiment 1 we measured CORT plasma levels to see if EA regulates CORT secretion. Rats were divided into four groups (n = 8 per group): CFA (0.08 ml) + EA, CFA + sham EA, CFA only, and EA only. EA was applied at 10 Hz, 3 mA, 0.1 ms pulse width for two 20 min periods, once at the beginning and once at the end of a 2 h period starting immediately after CFA injection. Blood (0.5 ml) was taken from each rat at baseline (before inflammation and/or EA) and 2 h and 5 h after inflammation. In Experiment 2 adrenalectomy (ADX) rats were used to study the effects of the adrenal gland on the therapeutic actions of EA. Rats were divided into two groups (n = 7 per group): 1) ADX rats + CFA (0.03 ml) + EA, 2) ADX rats + CFA + sham EA. The degree of edema, indicative of the intensity of inflammation, was quantified 2 h and 5 h after CFA injection into a hind paw by measuring paw thickness with a Laser Sensor (AR200–50, Acuity, Portland, OR). A paw withdrawal latency (PWL) test was conducted at the same time points. The investigator who conducted the measurement was blinded to the treatment assignments. Experiment 3 was to determine whether a prototypical glucocorticoid receptor antagonist, RU486, affects EA anti-edema. CFA (0.08 ml)-inflamed rats were divided into four groups (n = 7 per group): RU486 (Sigma) + sham EA, vehicle + sham EA, RU486 + EA and vehicle + EA. Paw thickness was measured as above. A 15 μg/μl concentration of RU486 was dissolved in Dimethyl Sulfoxide (DMSO), and 15 μl of RU486 or vehicle was injected locally into one hind paw 15 min before CFA injection.

### 2.3 Intravenous cannulation and blood sample collection

For intravenous cannulation, animals were anesthetized with sodium pentobarbital (50 mg/kg) intraperitoneally (i.p.) and surgically implanted with a chronic indwelling jugular catheter (Braintree Scientific, Inc). The catheter was secured with Mersilene surgical mesh (General Medical, New Haven, CT) at the jugular vein. It ran subcutaneously and exited at the animal's back through a 22-gauge tubing secured with mesh. Antibiotic ointment was applied to the wound. To prevent clogging, catheters were flushed every third day with 0.15 ml of gentamicin (120 μg/ml). At baseline and 2 h and 5 h post-CFA 0.5 ml of blood was withdrawn, and the lost volume was replaced with an equal volume of saline. Blood was centrifuged (1310 g) for 15 min at 4°C. The plasma was collected and stored at -80°C until assayed. CORT levels were measured with a commercially available ELISA kit (Cayman Chemical, MI) using the procedure recommended by the manufacturer. The detection limit of the kit is 24 ng/ml. The antibody in the kit specifically reacts with CORT and has less than 1% cross-reactivity with other adrenal hormones (e.g. aldosterone and cortisone). CORT concentrations (ng/ml) were determined by comparing samples to the standard curve generated with the kit.

### 2.4 ADX Surgery

Bilateral ADX surgery was performed via the dorsal approach under anesthesia with sodium pentobarbital (50 mg/kg, i.p.). The extracted tissue was dissected to confirm that both the adrenal cortex and adrenal medulla were removed completely. The rats were given 0.9% saline ad libitum to compensate for sodium loss after the operation and allowed to recover for five days before experimentation. Blood was withdrawn and CORT was measured as above on day 7 post-ADX.

### 2.5 Inflammation and hyperalgesia testing

Inflammation was induced by injecting CFA (0.5 mg/ml heat-killed *Mycobacterium tuberculosis *suspended in an 1:1 oil/saline emulsion; Sigma, St. Louis, MO) subcutaneously into the plantar surface of one hind paw of each rat using a 25-gauge hypodermal needle [20]. Inflammation appeared within 2 h of the injection and peaked between 6–24 h. PWL was tested with a previously described method [20,21]. Each rat was placed under an inverted clear plastic chamber on the glass surface of the Paw Thermal Stimulator System (UCSD, San Diego) and allowed to acclimatize for 30 min before the test. A radiant heat stimulus was applied to the plantar surface of each hind paw from underneath the glass floor with a projector lamp bulb (CXL/CXR, 8 V, 50 W). PWL to the nearest 0.1 sec was automatically recorded when the rat withdrew its paw from the stimulus. Stimulus intensity was adjusted to derive an average baseline PWL of approximately 10.0 s in naive animals. Paws were alternated randomly to preclude "order" effects. A 20-sec cut-off was used to prevent tissue damage. Mean PWL was established by averaging the latency of four tests with a 5-min interval between each test.

### 2.6 Acupuncture Treatment Procedures

To maximize the anti-inflammatory effect and to treat animals prophylactically, the EA treatment was given twice, for 20 min each, once immediately after the administration of CFA and again 2 h post-CFA [10]. EA parameters of 10 Hz, 3 mA, 0.1 ms pulse width, which showed significant anti-inflammatory and anti-hyperalgesic effects on the rat inflammation model in our previous studies [9,10], were used in the present study.

The equivalent of the human acupoint Huantiao (GB30) was chosen for bilateral needling based on traditional Chinese medicine (TCM) meridian theory [6], on its successful use in our previous studies, and on studies by others [10,22,23]. Based on our previous point-specificity study, EA produced better anti-hyperalgesia at GB30 than at acupoint Waiguan (the fifth acupoint on the Triple Energizer Meridian, TE 5) on the forepaw or at two non-specific points, an abdominal point and a point on the quadriceps opposite to GB30 [10]. Waiguan is located dorsally between the radius and ulna, 2 units (based on the standard acupuncture measurement of 12 units between the transverse cubital crease and the transverse wrist crease) above the transverse crease of the wrist. Underneath are the posterior interosseous nerve and the anterior interosseous nerve. In humans, GB30 is located at the junction of the lateral 1/3 and medial 2/3 of the distance between the greater trochanter and the hiatus of the sacrum; underneath are the sciatic nerve, inferior gluteal nerve and gluteal muscles [5]. GB30 was located on the rat's hind limbs using the comparable anatomical landmarks. After cleaning the skin with alcohol swabs, a disposable acupuncture needle (gauge # 32, 0.5 in long) was inserted obliquely approximately one half inch deep into GB30 on each of the animal's hind limbs, and a pair of electrodes was attached to the ends of the needles. The needles and the electrodes were stabilized with adhesive tape.

EA stimulation was delivered by an electrical stimulator (A300 Pulsemaster, World Precision Instruments) via an isolator (A360D Stimulus Isolator, World Precision Instruments) which converts electrical voltage into electrical current. This bilateral, cross-limb connection has been used previously by our team and others with no adverse effects [10,24], and although similar connections are frequently used in clinic, no adverse effects have ever been reported [25,26]. While EA frequency was held constant, intensity was adjusted slowly (over the period of approximately 2 min) to the designated level of 3 mA, which is the maximum EA current intensity that a conscious animal can tolerate [10]. Mild muscle twitching was observed. During EA treatment, each rat was placed under an inverted clear plastic chamber (approximately 5" × 8" × 11") but was neither restrained nor given anesthetic. The animals remained awake and still during treatment and showed no observable signs of distress. For the sham treatment control, acupuncture needles were inserted bilaterally into GB30 without electrical stimulation or manual needle manipulation. This sham procedure produced no anti-hyperalgesic effect on this animal model in our previous study [10]. Because it is most comparable to the treatment procedure but produces little therapeutic effect, we used it as sham control in this study.

### 2.7 Data Analysis

For clarity, plasma CORT level data were presented as changes in percentage: [(level _2–5h _-level_baseline_)/level_baseline_] × 100%. Paw thickness data were presented as mean ± SE. PWL data were presented as (PWL _2–5h _– PWL _baseline_)/PWL_baseline _*100%. Data were analyzed using analysis of variance (ANOVA) with repeated measures followed by *post-hoc *Tukey's multiple comparisons (GraphPad InStat). P < 0.05 was set as the level of statistical significance.

## 3. Results

### 3.1 EA increased plasma levels of CORT

**Figure 1:**
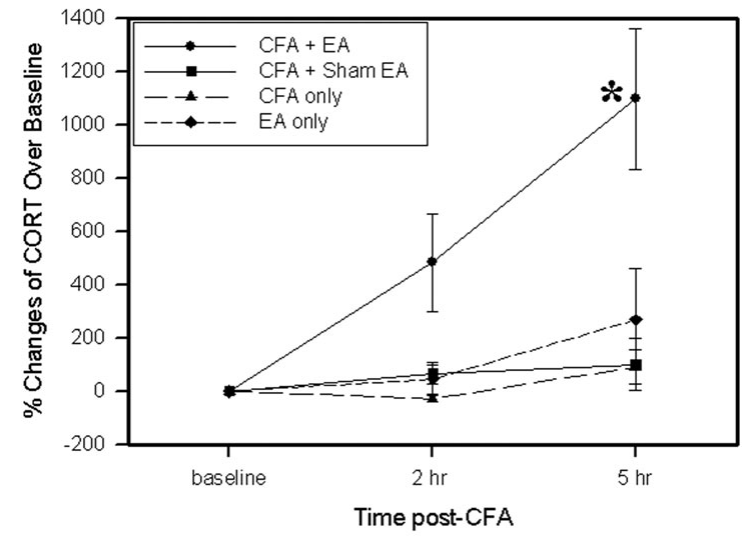
Effects of EA treatment on plasma levels of CORT. The data were presented as % changes vs baseline (n = 8 per group, mean ± SEM). EA treatment in non-inflamed rats (diamond) induced no significant changes of plasma CORT. CFA-induced inflammation alone (triangle) resulted in no significant changes in plasma CORT levels. EA treatment in inflamed rats (circle) significantly increased CORT levels compared to sham EA (square). *P < 0.05 compared sham EA.

As shown in Fig. 1, plasma CORT levels in EA-treated inflamed rats were significantly higher at 2 and 5 h post-CFA injection than those in sham-treated inflamed rats, the plasma CORT levels of which did not significantly change compared to base levels during the same period. The data indicate that EA effects on CORT may last at least 3 h. In contrast to CFA-inflamed rats, EA treatment of non-inflamed rats produced no significant changes in plasma CORT level. CFA-induced inflammation alone did not caused significant plasma CORT changes.

### 3.2 ADX and RU486 blocked EA anti-edema

**Figure 2:**
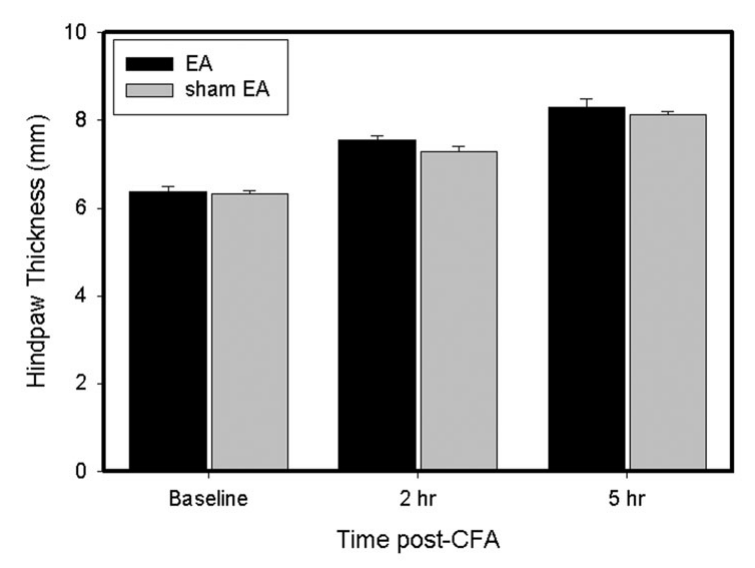
EA effects on edema in ADX rats. The data were presented as mean ± SEM. All rats (n = 7 per group) were CFA-inflamed. Note that EA did not inhibit edema in ADX rats. Edema was determined by increased paw thickness (mm).

We previously reported that EA significantly inhibits edema compared to sham EA control in CFA-injected rats [9]. Fig. 2 shows that hind paw thickness was the same in EA-treated and sham EA-treated CFA-injected ADX rats. That EA had no effect on edema in ADX rats indicates that ADX abolished the anti-edema effect of EA. In ADX rats, plasma CORT significantly (P < 0.05) decreased from baseline 7 days post-ADX, from 5087.85 ± 1017.64 to 394.78 ± 103.14 ng/ml.

**Figure 3:**
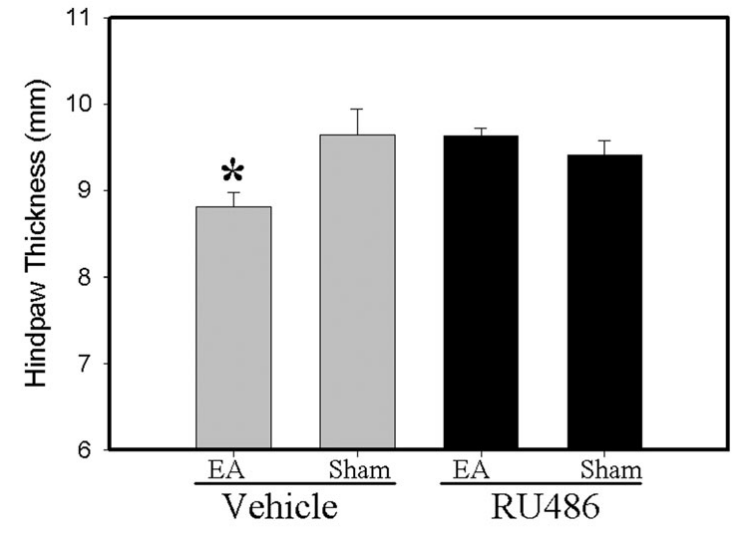
Effects of RU486 on EA anti-edema in CFA-inflamed rats. The data were presented as mean ± SEM. The hind paw thickness is the average of values at 2 h and 5 h and was analyzed with two-way ANOVA (n = 7 per group). EA + vehicle (column 1) significantly alleviated edema compared to sham + vehicle control (column 2). After RU486 pretreatment (columns 3 & 4), EA showed no anti-edema effects (column 3) compared to sham control (column 4). * p < 0.05 compared to sham EA.

In the RU486 study (Fig. 3.), paw thickness in EA-treated rats plus vehicle was significantly less than that in sham EA-treated rats plus vehicle, suggesting that EA significantly inhibited edema compared to sham control. However, paw thickness in EA-treated rats plus RU486 was no different from that in sham EA rats plus RU486, indicating that RU486 blocked EA anti-edema.

### 3.3 ADX did not abolish EA anti-hyperalgesia

**Figure 4:**
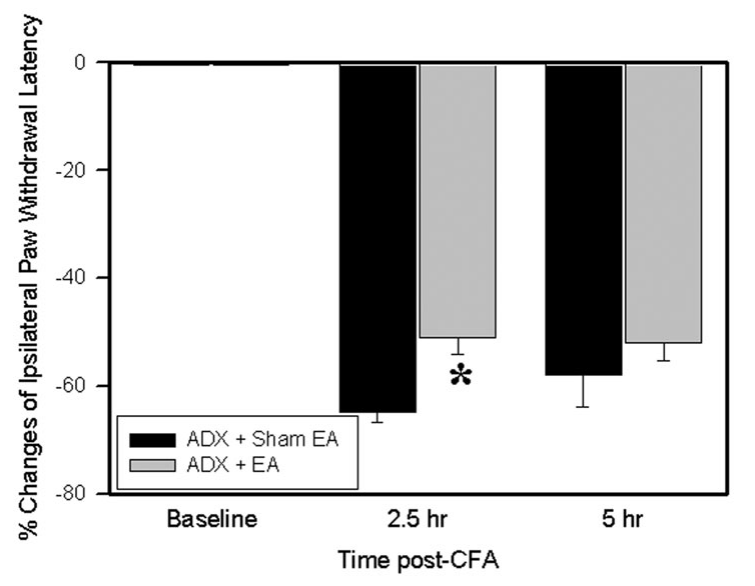
EA effects on hyperalgesia in ADX rats. The data were presented as mean ± SEM. All rats (n = 7 per group) were inflamed with CFA. Note that EA significantly inhibited hyperalgesia at 2.5 h post-CFA in ADX rats. Hyperalgesia was defined as a decrease in PWL (sec). * p < 0.05 compared to sham EA.

Fig. 4 shows the effects of ADX on EA-produced anti-hyperalgesia. Before the CFA, overall mean baseline PWL to noxious heat stimuli was similar in all groups of rats, and there was no significant PWL difference between left and right hind paws. Following an injection of 0.03-ml CFA, PWL of the injected paw was significantly less than that of the contralateral hind paw, which was unchanged from baseline. EA treatment significantly (P < 0.05) inhibited hyperalgesia 2 h post-CFA compared to sham EA in ADX rats.

## 4. Discussion

The main findings of the present study are that EA activates the adrenal glands and increases endogenous CORT to suppress inflammation. We found that EA significantly increased plasma CORT levels in CFA-inflamed rats compared to sham EA. Because CORT is mainly secreted by the adrenals and has potent anti-inflammatory effects, an increase of plasma CORT after EA treatment suggests that EA activates the adrenals to suppress CFA-induced edema. This is further evidenced by our experiments with ADX rats and the glucocorticoid receptor antagonist, RU486. Bilateral ADX completely blocked any EA-produced anti-edema effect. Pretreatment with RU486 also prevented EA anti-edema. Taken together, these results suggest that EA activates the adrenals in pathological conditions to increase glucocorticoid secretion and suppress inflammatory responses.

We also found that EA increased plasma CORT levels in inflamed rats but did not affect levels in naive rats. Hypothetically, stress responses during EA treatment might be stronger in inflamed animals than in naive animals. However, we exclude this possibility because, as we reported previously [9], the same stimulation intensity at acupoint GB30 as that used in this study did not change heart rate or blood pressure, both of which are indicators of stress response, in CFA-inflamed animals. This demonstrates that EA procedures and acupuncture needle stimulation do not induce significant stress responses. Further, the sham EA induced no changes in the plasma CORT levels of inflamed rats, indicating that the EA procedures used in this study have no stress effects. Thus we believe that the CORT level increase in EA-treated CFA rats is not a general stress response. The discrepancy between inflamed and naive rats in response to the same EA treatment suggests that EA affects healthy and pathological conditions differently. This is supported by a previous study reporting that naloxone completely blocked EA analgesia in healthy rats but partly blocked EA anti-hyperalgesia in carrageenan-induced inflammatory rats, and EA analgesia lasted longer in inflamed rats than in healthy rats [19]. Clinically, acupuncture produces long-term (days) pain relief in patients [27], while it produces short-term (minutes) analgesia [28] in healthy humans. Moreover, acupuncture significantly decreases the systolic pressure in patients with hypertension, but it did not exert significant influence on either systolic or diastolic pressure in patients with normal blood pressure [29]. Collectively, these studies corroborate our finding that EA works differently in healthy than in pathological conditions. Others have reported, however, that acupuncture increased serum levels of cortisol in healthy humans and horses [13,14]. This discrepancy may be due to differences in the subjects used in the studies.

It should be noted that bilateral ADX did not block EA anti-hyperalgesia, suggesting that endogenous CORT plays little part in EA anti-hyperalgesia. Consistent with this, a previous study reported that endogenous CORT does not reduce nociception in formalin-induced pain model [30]. This suggests that EA may produce anti-hyperalgesia by affecting the nervous system. Our previous studies demonstrated that spinal mu and delta opioid systems are involved in EA anti-hyperalgesia in the same inflammatory pain rat model [20].

## 5. Conclusion

The present study demonstrates that EA significantly increases plasma CORT levels and suppresses edema by activating the adrenal glands. It also suggests that EA produces different effects in healthy and in pathological conditions.

## Competing interests

The authors declare that they have no competing interests.

## Authors' contributions

AL and RZ designed the study, carried out the animal surgery, acupuncture treatment and blood collections, and drafted the manuscript. YW and HZ carried out the behavioral tests. KR, BMB and LL participated in the design and helped draft the manuscript. MT performed the statistical analysis. All authors read and approved the final manuscript.

## Pre-publication history

The pre-publication history for this paper can be accessed here:


